# High Occurrence of Zoonotic Subtypes of *Cryptosporidium* *parvum* in Cypriot Dairy Farms

**DOI:** 10.3390/microorganisms10030531

**Published:** 2022-02-28

**Authors:** Sumaiya Hoque, Daphne E. Mavrides, Pedro Pinto, Silvia Costas, Nisa Begum, Claudia Azevedo-Ribeiro, Maria Liapi, Martin Kváč, Stavros Malas, Eleni Gentekaki, Anastasios D. Tsaousis

**Affiliations:** 1 Laboratory of Molecular and Evolutionary Parasitology, RAPID Group, School of Biosciences, University of Kent, Canterbury CT2 7NJ, UK; sh986@kent.ac.uk (S.H.); pp410@kent.ac.uk (P.P.); silvia.costas24@hotmail.com (S.C.); nisabegum@hotmail.co.uk (N.B.); c.azevedo-ribeiro@kent.ac.uk (C.A.-R.); 2 Department of Basic Sciences, University of Nicosia Medical School, Nicosia 2408, Cyprus; mavrides.d@live.unic.ac.cy (D.E.M.); malas.st@unic.ac.cy (S.M.); 3 Veterinary Services of Cyprus, Nicosia 1417, Cyprus; mliapi@vs.moa.gov.cy; 4 Biology Centre CAS, Institute of Parasitology, Czech Academy of Sciences, 370 05 České Budějovice, Czech Republic; kvac@paru.cas.cz; 5 Faculty of Agriculture, University of South Bohemia, 370 05 České Budějovice, Czech Republic; 6 School of Science, Mae Fah Luang University, Chiang Rai 57100, Thailand; gentekaki.ele@mfu.ac.th; 7 Gut Microbiome Research Group, Mae Fah Luang University, Chiang Rai 57100, Thailand

**Keywords:** *Cryptosporidium*, *Cryptosporidium parvum* detection, subtyping, *gp60*, 18S rRNA, calves, Cyprus, zoonosis

## Abstract

*Cryptosporidium parvum* is one of the major causes of neonatal calf diarrhoea resulting in reduced farm productivity and compromised animal welfare worldwide. Livestock act as a major reservoir of this parasite, which can be transmitted to humans directly and/or indirectly, posing a public health risk. Research reports on the prevalence of *Cryptosporidium* in ruminants from east Mediterranean countries, including Cyprus, are limited. This study is the first to explore the occurrence of *Cryptosporidium* spp. in cattle up to 24 months old on the island of Cyprus. A total of 242 faecal samples were collected from 10 dairy cattle farms in Cyprus, all of which were screened for *Cryptosporidium* spp. using nested-PCR amplification targeting the small subunit of the ribosomal RNA (*18S* rRNA) gene. The 60 kDa glycoprotein (*gp60*) gene was also sequenced for the samples identified as *Cryptosporidium parvum*-positive to determine the subtypes present. The occurrence of *Cryptosporidium* was 43.8% (106/242) with at least one positive isolate in each farm sampled. *Cryptosporidium bovis, Cryptosporidium ryanae* and *C. parvum* were the only species identified, while the prevalence per farm ranged from 20–64%. Amongst these, the latter was the predominant species, representing 51.8% of all positive samples, followed by *C. bovis* (21.7%) and *C. ryanae* (31.1%). Five *C. parvum* subtypes were identified, four of which are zoonotic—IIaA14G1R1, IIaA15G1R1, IIaA15G2R1 and IIaA18G2R1. IIaA14G1R1 was the most abundant, representing 48.2% of all *C. parvum* positive samples, and was also the most widespread. This is the first report of zoonotic subtypes of *C. parvum* circulating in Cyprus. These results highlight the need for further research into the parasite focusing on its diversity, prevalence, host range and transmission dynamics on the island.

## 1. Introduction

*Cryptosporidium* is an apicomplexan parasite and the causative agent of cryptosporidiosis [1]. Clinical signs of this disease include watery diarrhoea and dehydration, which can prove fatal, particularly in immunocompromised individuals and infants [2,3]. The transmission form of the parasite is the oocyst, which can be transmitted directly via the faecal–oral route or indirectly through contaminated food and water [1]. Oocysts are very robust, are highly resistant to environmental changes and can remain dormant for up to six months [1,4]. Millions of oocysts can be shed through bowel movements of infected hosts, both humans and other animals. Coupled with a lack of effective therapeutic agents and vaccines [5,6], it has proven difficult to manage outbreaks and the spread of *Cryptosporidium*. The outbreaks are commonly of a zoonotic nature with farms constituting a major reservoir of the parasite [7].

Of the 49 described *Cryptosporidium* species, around 20 of them are zoonotic [8,9]. Molecular surveillance of *Cryptosporidium* ssp. outbreaks indicates that the zoonotic *C. parvum* and the anthroponotic *C. hominis* are responsible for the majority of human cryptosporidiosis cases [10,11]. Amongst farm animals, goats and cattle are the main hosts of the organism. In cattle farms, *C. andersoni*, *C. bovis*, *C. ryanae* and *C. parvum* are the major species. Age-related variance of infecting *Cryptosporidium* species has been shown, with *C. parvum* typically infecting neonatal calves, while the others are typically found in adults, heifers and post-weaned calves [12]. *Cryptosporidium parvum* is the most clinically significant as it is the most widespread species in cattle [13,14,15] and a major cause of enteritis, causing neonatal diarrhoea and subsequent long-term adverse effects on weight gain [6,13,16]. The disease leads to significant economic losses for cattle farmers due to reduced production efficiencies of meat and milk [17,18,19]. Aside from the financial impact of *Cryptosporidium* infections, cattle have been well-established as a key source of zoonotic cryptosporidiosis, giving an epidemiological dimension to the issue [5,20,21].

Prevalence investigations have frequently been carried out in almost all continents [22,23]. Studies of *Cryptosporidium* occurrences in Europe point towards a high prevalence [24,25,26]. These investigations have primarily focused on mainland Europe, while island nations have been largely overlooked—with the exception of the UK [27]. Island settings are ideal for studying the epidemiological and ecological aspects of parasitic organisms due to restricted boundaries and the subsequent limited movement of hosts. Regrettably, there is a lack of information on the prevalence and subtypes of *Cryptosporidium* spp. circulating in small European island nations.

This study provides the first molecular epidemiological data on *Cryptosporidium* spp. in dairy cattle in Cyprus. The island is the third largest in the Mediterranean region and is in the paths of three continents. Using molecular techniques and analyses, we were able to identify *Cryptosporidium* species and subtypes circulating within the examined cattle farms. This work will spearhead further studies on the roles and effects of the parasite in this unique region of the world.

## 2. Methods

### 2.1. Study Area

**Figure 1:**
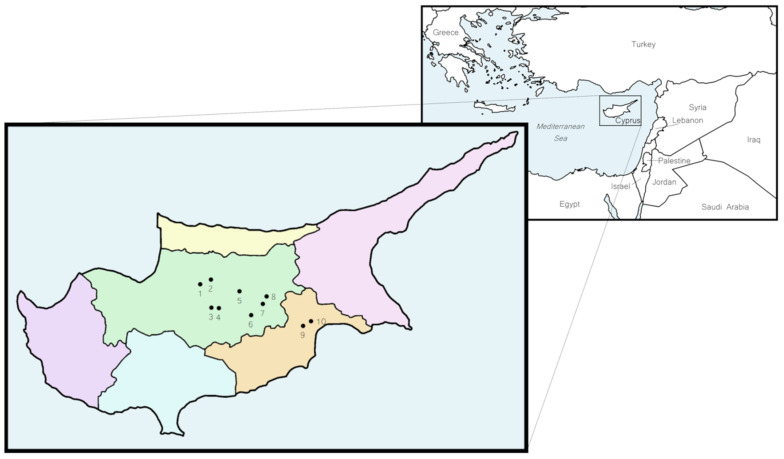
Geographic location of Cyprus in the East Mediterranean region and distribution of the cattle farms sampled. Farms 1–8 were located across the Nicosia district (green), while farms 9–10 were located in the Larnaca district (orange).

Cyprus has 358 dairy cattle farms with approximately 43,900 cows of the Holstein Friesian breed. All cattle farms in Cyprus are intended for dairy production as there are no farms that specifically breed for meat production. Farms are located in all five districts of Cyprus: Nicosia, Larnaca, Limassol, Ammochostos and Paphos. Samples were collected from ten randomly selected farms located in Larnaca and Nicosia (Figure 1). These two districts were chosen as this is where most farms are located, many of which are heavily aggregated with a close proximity between them. Eight farms were located in Nicosia and two were located in Larnaca. The Nicosia district lies in the centre of the island, and samples were taken from the Dali, Tseri, Ayia Varvara, Akaki and Arediou regions. Most of the selected farms in Nicosia are at a relatively low altitude (200–400 m), experiencing hot, dry and humid summers and cold winters with minimal precipitation. The two farms in the Larnaca district were located in the Aradippou region, which has a great confluence of farms. The area experiences a climate similar to that of Nicosia with a slightly elevated humidity due to a closer proximity to the sea and a lower altitude (80 m). Farmers were invited to participate in the study by the employed veterinarian, who also explained the research project and its overall aims and objectives. The study was under the umbrella of the National Veterinary Services. Cows from all selected farms were of the dairy-producing Holstein Friesian breed. Most farms in Cyprus have intensive to semi-intensive farming conditions (semi-intensive is a system where cattle are exposed to a combination of intensive and extensive methods, which can alternate year-round depending on the weather). Cows are usually housed indoors year-round to avoid heat stress due to warm weather and a lack of grass for grazing outdoors. Rainfall is minimal and so irrigation is necessary to compensate for limited water resources. All participating farms in this study raised their cows semi-intensively.

### 2.2. Sample Collection and DNA Extraction

A total of 242 faecal samples were collected in November 2019. While the study focused mainly on pre-weaned animals, samples were also collected from animals up to two years of age (see Supplementary Table S1). Animals were randomly selected. Faeces were collected immediately after defecation, placed in sterile tubes and stored on ice. Tubes were stored at −20 °C upon arrival at the laboratory. Throughout a random sampling, a mix of diarrheic and asymptomatic calves were sampled (depending on the age of the calves; samples from younger calves were mostly in mushy to liquid forms than the samples from older calves). The condition of the faeces was noted using the Bristol stool score (see Supplementary Figure S1), with higher scores representing softer, watery stools. DNA extraction was carried out using 200 mg of faeces per sample and the PureLink™ Microbiome DNA Purification Kit (Thermo Fisher Scientific, Carlsbad, CA, USA) according to the manufacturer’s instructions, with slight modifications. Specifically, 650 μL of S1 lysis buffer was used for each sample. After addition of the S2 lysis enhancer, the samples were incubated for 13 min at 65 °C and homogenised for a further 13 min. Following an addition of the S3 clean-up buffer, the samples were incubated at 4 °C for 10 min to optimise the removal of proteins. After an addition of 100 μL of the S6 elution buffer, the samples were incubated at room temperature for 3 min before centrifugation to improve DNA yield. Genomic DNA was stored at −20 °C until amplification reactions of the small subunit ribosomal RNA (*18S* rRNA) and 60 kDa glycoprotein (*gp60*) gene were carried out.

### 2.3. Cryptosporidium spp. Screening and Molecular Genotyping

Samples were screened for *Cryptosporidium* spp. using nested-PCR amplification of the 631 bp region of the *18S* rRNA gene [24,28]. Positive (genomic DNA from a pure culture of *C. parvum*) and negative (water was used as template instead of DNA) controls were included in both the reactions. PCR products were separated on a 2% gel and extracted using the GeneJET Gel Extraction Kit (Thermo Fisher Scientific, Carlsbad, CA, USA). Sanger sequencing was used to identify *Cryptosporidium* spp. Samples were bidirectionally sequenced (Eurofins Genomics, Wolverhampton, UK) using internal PCR primers. Chromatograms were manually assessed for quality and ambiguous bases were trimmed on both ends of the reads. For species-level identifications, sequences were used as queries to perform BLAST searches against the nucleotide database in GenBank, followed by an alignment with reference sequences.

### 2.4. Cryptosporidium Screening and gp60 Subtyping

To determine the subtypes of the *Cryptosporidium 18S* rRNA PCR-positive samples, a nested PCR of the *gp60* gene was carried out [24,29]. Positive and negative controls were as described above and included in both reactions. PCR products were separated on a 2% gel and extracted using the GeneJET Gel Extraction Kit (Thermo Fisher Scientific, Carlsbad, CA, USA). Sanger sequencing was used to identify *Cryptosporidium* subtypes. Samples were bidirectionally sequenced (Eurofins Genomics, Wolverhampton, UK) using the internal PCR primers. Chromatograms were manually assessed for quality and ambiguous bases were trimmed on both ends of the reads. Subtypes were determined using established standard nomenclature [30]. Newly generated sequences were used as queries to perform BLAST searches against the nucleotide database in GenBank, followed by an alignment with reference sequences. Polymorphisms were identified using these alignments.

## 3. Results

### 3.1. Cryptosporidium spp. Occurrences across Cypriot Farms

The amplification of the *18S* rRNA gene showed an occurrence of 43.8% with 106/242 specimens positive for *Cryptosporidium* spp. (Table 1). Occurrences varied across the farms from 20% (3/15) to 64% (16/25).

The majority of the positive samples were identified as *C. parvum* (47.2%, 50/106), with 41 of them showing a 100% nucleotide identity to the reference sequence AH006572.2 and one sample showing a 99% nucleotide identity to the same sequence. We were unable to obtain good quality sequences for eight *18S* rRNA PCR-positive samples. Nonetheless, a *C. parvum* identity was confirmed through a positive *gp60* PCR and subsequent sequencing. At the farm level, *C. parvum* was present in 9/10 farms, with occurrences ranging from 9.1% to 34.8%.

The next most common species present was *C. ryanae* (25.5%, 27/106). Additionally, six samples from three farms had co-infections of *C. ryanae* and *C. parvum*, with their presence determined through *18S* rRNA and *gp60* amplifications, respectively. Twenty-nine samples had a 100% nucleotide identity to the reference sequence KF128756.1, while one was 99% identical to the same sequence. Another variant of *C. ryanae* was identified, with three samples having a 100% nucleotide identity to the reference sequence KT922233.1. *Cryptosporidium bovis* was the least prevalent species identified (21.7%, 23/106), with all 23 samples showing a 100% nucleotide identity to the reference sequence EU827363.2. Representative nucleotide sequences of *18S* rRNA have been deposited in GenBank under the accession numbers OL348064-OL348160.

### 3.2. Cryptosporidium parvum Subtyping through gp60 Analysis

All 50 of the *18S* rRNA PCR-positive samples were screened using a nested PCR of the *gp60* gene. Of these, the *gp60* gene was successfully amplified and sequenced in 42 samples and then subtyped for *C. parvum*. A further five *gp60*-positive samples were identified as *C. ryanae*. Sequence analysis revealed the presence of five subtypes, all belonging to the IIa *C. parvum* family (Table 2). Nine of the farms sampled contained at least one *C. parvum* subtype.

The IIaA14G1R1 subtype was the most numerically prevalent, occurring in 60% of the farms, making it also the most widely distributed. It was the sole subtype present on three farms and represented 57.4% (27/47) of all the *C. parvum*-positive samples with successful *gp60* sequencing. All 27 showed a 100% nucleotide identity to the reference sequence MN815774.1. The IIaA12G1R1 subtype was the next-most prevalent and was only found in one farm, accounting for 29.7% (14/47) of all the *C. parvum* infections. All 14 samples were identical and showed a 99% nucleotide identity to the reference sequence MW411017.1. Four samples were identified as IIaA15G2R1, with two of them showing a 100% nucleotide identity to the reference sequence DQ630518.1, while the other two were 99% identical with the same sequence. One sample was identified as IIaA15G1R1, with a 99% nucleotide identity to the reference sequence AB777872.1, and another was identified as IIaA18G2R1, with a 99% nucleotide identity to the reference sequence DQ630515.1 (Table 3). Representative nucleotide sequences of *gp60* have been deposited in GenBank under the accession numbers OL462897-OL462943.

### 3.3. Geographical Distribution of Subtypes

**Figure 2:**
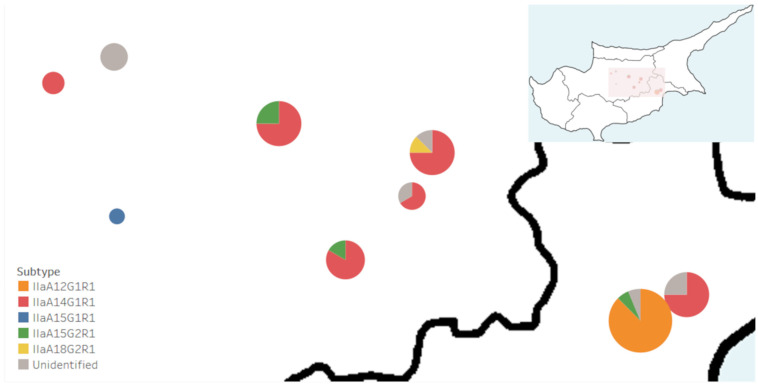
*Cryptosporidium parvum* subtype prevalence across Cypriot cattle farms. Geographical distribution of *gp60* subtypes across Cyprus cattle farms: IIaA12G1R1 (orange), IIaA14G1R1 (red), IIaA15G1R1 (blue), IIaA15G2R1 (green) and IIaA18G2R1 (yellow). *C. parvum*-positive samples with unsuccessful gp60 sequencing are indicated in grey. Pie charts are proportional to number of *C. parvum*-positive samples identified per farm.

For easy visualization, subtype names were also indicated by their colour as depicted in Figure 2. The most broadly distributed subtype was IIaA14G1R1 (red), present in 6/9 (67%) of the *C. parvum*-positive farms, followed by IIaA15G2R1 (green), present in 3/9 (33%) farms. In all cases, the IIaA15G2R1 (green) subtype co-occurred with another and was the least dominant of the two. In two cases, a co-occurrence was with IIaA14G1R1 (red), and in one case, a co-occurrence was with IIaA12G1R1 (orange). The latter subtype, along with IIaA18G2R1 (yellow) and IIaA15G1R1 (blue), occurred only in one farm each. In four farms, only a single subtype was detected: IIaA14G1R1 (red) in three and IIaA15G1R1 (blue) in one. Notably, in Larnaca, the two sampled farms were in very close proximity, but the subtypes present did not overlap. One farm had only the IIaA14G1R1 (red) subtype, while the other had IIaA12G1R1 (orange) and IIaA15G2R1 (green).

## 4. Discussion

This is the first study to investigate *Cryptosporidium* spp. occurrences in the dairy farms of Cyprus. The study mainly focused on young calves up to three months of age, although we have collected samples from older calves as well. The overall occurrence of the parasite was 43.8%. Previous molecular studies on the surrounding Mediterranean region have generally reported lower incidences of *Cryptosporidium* infections amongst cattle. Specifically, studies using *18S* rRNA PCR and sequencing in Turkey found prevalence ranges of 3.9–53.6% [31,32,33,34,35]. In Egypt, the subtype-based prevalence was between 7 and 30.2% [36,37,38,39,40]. A small-scale PCR-based study in Jordan found 18.7% of asymptomatic cattle carried the organism [41]. The discrepancy in the prevalence observed among these studies may be due to various factors, including the ages of the cows, the farm/herd locations, seasonality, farm management and methods of detection. Due to time and financial constraints, microscopic analysis of faeces is a popular screening method, where only microscopically positive samples undergo molecular genotyping. While these studies do provide information on the *Cryptosporidium* species and *C. parvum* subtypes present, they likely underestimate prevalence due to the reduced sensitivity of microscopy as a screening technique. Additionally, some studies only targeted diarrheic animals [33,35], ignoring the presence of *Cryptosporidium* in asymptomatic individuals. The presence of the parasite in non-diarrheic samples is still of clinical significance as asymptomatic animals can still shed oocysts in their environment and spread infection [6]. Herein, we used a nested-PCR approach to identify and subtype (see below) *Cryptosporidium*, which is a more sensitive and specific method. At the farm level, *Cryptosporidium* was broadly distributed across all farms. This finding matches previous flock-level studies in goats and sheep in Cyprus [42]. Even though this is a preliminary finding, it shows that the parasite is widespread in this region, hinting at its potential of zoonotic transmission. Follow-up studies should expand to include farms across the whole island. The variations in *Cryptosporidium* occurrences per farm could indicate differences in farm management practices. Climate variables, such as temperature and precipitation, may also play a part in the *Cryptosporidium* spread [43,44], with some studies showing seasonal variations in *Cryptosporidium* prevalence in cattle farms [45]. Nonetheless, in this study, there was no precipitation, and there was a minimal variation in temperature in the region during the sampling period (see Supplementary Figure S2). As such, future investigations should report these variables along with potential risk factors, e.g., calf husbandry practices, box dynamics, bedding, colostrum feeding schedules, climate, and geographical variables, to understand their impacts on parasite spreading. A shortcoming of this study is the lack of information that allows for the stratification of age data for the sampled cows. As *C. parvum* has been well established to occur most often in neonatal calves up to three weeks old, it is possible the differences in the prevalence between farms were due to differences in the age ranges of the cows sampled.

*Cryptosporidium parvum* was the most numerically predominant species present, representing 51.8% of total infections, followed by *C. ryanae* at 31.1% and *C. bovis* at 21.7%. Our results on the predominance of *C. parvum* are in agreement with those from surrounding regions on both healthy and diarrheic animals [31,35,36,46,47,48]. The predominance of *C. parvum* specifically in pre-weaned cattle has also been reported in the USA [12]. At the farm level, *C. parvum* and *C. ryanae* were found in 90% of the farms, while *C. bovis* was present in 60%. *C. ryanae* and *C. bovis* infections are typically asymptomatic and occur in older animals. Since this study mainly focused on pre-weaned and young calves, it is possible that these two species might be under-represented. Hence, future studies should focus on a wider age range of cattle.

Amongst the 55 *C. parvum*-positive samples identified with *18S* rRNA, including co-infections, only 47 were subtyped. The rest of the samples could not be subtyped due to unclear sequence chromatograms. This could suggest that the calves from which the samples were obtained carried multiple subtypes of *C. parvum*. A drawback of nested PCR and Sanger sequencing is the amplification and detection of only the more abundant species in a sample, while mixed or less abundant species are essentially hidden from detection. Previous molecular studies on *Cryptosporidium* have employed next-generation sequencing and have successfully identified mixed infections as well as less abundant species and subtypes [49]. Recently, TIDE, a new bioinformatics platform, was used to discern the multiple *gp60* subtypes present in obscure chromatograms [50]. Hence, a combination of metagenomics and upcoming methodologies will assist in tackling this common issue in the future.

As is typical in cattle, all the subtypes were in the IIa family; however, the predominant *C. parvum* subtypes found in this study do not correspond to those of surrounding countries. For instance, in Turkey, the endemic subtype is IIaA13G2R1. In Egypt, the IId family is most commonly found in cows, with the IIdA20G1 subtype being endemic [37,38,39,40,46,51]. As IId subtypes typically infect smaller ruminants, such as lambs and goats, it is possible its prevalence in Egypt’s cattle is due to close contact with other livestock [52]. Herein, the most numerically abundant *C. parvum* subtype was IIaA14G1R1 (red colour, Figure 2), and it was also the most widespread, having been found in 6/10 farms. This subtype has been reported previously in cows from Turkey [31], Austria [53,54], Estonia [55], Poland [56], Germany [57] and the Netherlands [24], though it was typically less abundant in all cases. Outbreaks of human cryptosporidiosis in Norway and New Zealand [58,59] have been attributed to IIaA14G1R1, and the subtype has also been found in human samples from the USA [60], Ethiopia [61], Slovenia [62] and Slovakia [63]. In the UK, IIaA14G1R1 has been found in animal housing premises that have been identified as potential sources of transmission [64]. This is the first report on IIaA14G1R1 as the predominant subtype circulating in a region, highlighting its zoonotic potential. However, as this study had a relatively small sample size, the subtypes identified here may not be indicative of the entire cattle population in this country.

The IIaA12G1R1 (orange, Figure 2) subtype comprised 25.4% of the *C. parvum*-positive samples and was only identified in a single farm. Previously, IIaA12G1R1 has been detected in cows in Israel, making this report the second one in the region [47]. The zoonotic potential of this subtype remains to be determined, having so far only been reported in animals. The IIaA15G2R1 (green, Figure 2) subtype is the most predominant worldwide [65], including the neighbouring countries Turkey [66] and Israel [47]. However, it occurred at relatively low levels in the Cypriot farms studied (8.5%). IIaA15G2R1 is responsible for the majority of acute clinical diseases in humans; hence, its zoonotic potential has been well established [14,65]. Nonetheless, our results indicate it may not play a key role in zoonotic transmissions in Cyprus, though this needs further investigations with larger sample sizes.

Single isolates of IIaA15G1R1 (blue, Figure 2) and of IIaA18G2R1 (yellow, Figure 2) were also detected. In cattle, IIaA15G1R1 has been found in Egypt [36,39], Sweden [67] and the Czech Republic [68]. Its zoonotic potential appears to be high, having been linked to numerous instances of human cryptosporidiosis globally. For instance, in Scotland, IIaA15G1R1 is responsible for 47% of human cryptosporidiosis cases [69]. The subtype has also been identified in humans with diarrhoea who are from England [70], Australia [71], Egypt [46], Slovenia [62] and Lebanon [72]. IIaA18G2R1 has been previously identified in cattle in the USA [73], Northern Ireland [74], Germany [75], Italy [76] and France [24], though typically has been one of the less common subtypes found. This is a potentially zoonotic subtype having been identified sporadically in humans from England [70], the USA [60,77] and Australia [78].

## 5. Conclusions

To date, there have been no studies on human cryptosporidiosis in Cyprus [79]. Despite this, our study revealed a high occurrence of *C. parvum* (90%) in dairy calves, with four out of the five subtypes identified being zoonotic. This brings into question the circulation of the various subtypes not only in calves, but also in human and other animal hosts as well as the environment. It is worth noting that Cyprus imports cattle from Austria, the Czech Republic, Denmark, Germany, Italy, and the Netherlands for both breeding and production purposes. Hence, screening these animals for *Cryptosporidium* spp. would help track the circulation and introduction of various subtypes across the different countries. While this report represents a first step in determining *Cryptosporidium* burdens in cattle in Cyprus, significant literature gaps on the prevalence, transmission dynamics, sources of infection and effective interventions remain, calling into attention the need for a One Health approach in the immediate future.

## Figures and Tables

**Table 1:** *Cryptosporidium* spp. prevalence in Cypriot dairy farms.

Farm	No. of Specimens	Age Range	*Cryptosporidium* spp. Present			
			*C. parvum*	*C. bovis*	*C. ryanae*	*C. ryanae*/*C. parvum* Co-Infection
1	13	0–2 years	3	–	2	–
2	11	9–98 days	2	2	–	–
3	11	Pre-weaned	1	2	4	–
4	15	27–73 days	–	–	3	–
5	25	8–11 months	7	3	5	1
6	23	Pre-weaned	5	3	2	1
7	19	1–3 months	3	2	1	–
8	23	Pre-weaned	8	–	3	–
9	41	Pre-weaned	13	3	2	3
10	61	Pre-weaned	8	8	6	–
Overall	242		50	23	28	5

**Table 2:** Number of *Cryptosporidium parvum gp60* subtypes identified out of total *C. parvum*-positive samples per farm.

Farm	Subtypes (No. of Subtype/Total *C. parvum* Samples per Farm)
1	Unidentified (3/3)
2	IIaA14G1R1 (2/2)
3	IIaA15G1R1 (1/1)
4	–
5	IIaA14G1R1 (6/8), IIaA15G2R1 (2/8)
6	IIaA14G1R1 (5/6), IIa15G2R1 (1/6)
7	IIaA14G1R1 (6/8), IIaA18G2R1 (1/8), Unidentified (1/8)
8	IIaA14G1R1 (2/3), Unidentified (1/3)
9	IIaA12G1R1 (14/16) IIa15G2R1 (1/16), Unidentified (1/16)
10	IIaA14G1R1 (6/8), Unidentified (2/8)

**Table 3:** Polymorphisms in *Cryptosporidium 18S* SSU rRNA and *gp60* gene sequences showing intra-species genetic variability.

Gene	*Cryptosporidium* Species/Subtype	GenBank Accession Number	Polymorphisms ^b^	Reference Sequence
*18S*	*C. parvum*	OL348120	G→A, position 701	AH006572.2
*18S*	*C. ryanae*	OL348112	T insertion, position 490	KF128756.1
*gp60*	*C. parvum* (IIaA12G1R1)	OL462923 ^a^	A→G, position 183 C→T, position 721	MW411017.1
*gp60*	*C. parvum* (IIaA15G2R1)	OL462910	G→A, positions 163 and 581 A→T, position 639 A→G, position 687	DQ630518.1
*gp60*	*C. parvum* (IIaA15G2R1)	OL462917	T→C, positions 454 and 469	
*gp60*	*C. parvum* (IIaA15G1R1)	OL462922	C→T, position 753	AB777872.1
*gp60*	*C. parvum* (IIaA18G2R1)	OL462903	T→G, position 375	DQ630515.1

^a^ Though multiple identical sequences were found, only one accession number is given for simplicity; ^b^ positions indicate differences from the reference sequence.

## Data Availability

All data have been submitted to GenBank as shown in the methods section.

## Supplementary Materials

The following supporting information can be downloaded at https://www.mdpi.com/article/10.3390/microorganisms10030531/s1, Figure S1: Bristol stool score ranges for the *Cryptosporidium* spp. identified in this study, Figure S2: Meteorological report on Nicosia and Larnaca, indicating average temperature and rainfall in these regions during the sampling period, Table S1: Age range of sampled cows at each farm.
