# Supplementary material for: High Occurrence of Zoonotic Subtypes of Cryptosporidium parvum in Cypriot Dairy Farms
Source: Microorganisms. 2022 Feb 28;10(3):531. doi: 10.3390/microorganisms10030531 (PMC8951114; doi:10.3390/microorganisms10030531)
Supplement: Supplementary file 1 [file microorganisms-10-00531-s001.zip › microorganisms-1589894-supplementary.pdf]

microorganisms

Brief Report

High Occurrence of Zoonotic Subtypes of *Cryptosporidium parvum* in Cypriot Dairy Farms

Supplementary Materials

Table S1. Age range of sampled cows in each farm.

Farm	Age Range (Known Samples Only)
1	0–2 years
2	9–98 days
3	Pre-weaned
4	27–73 days
5	8–11 months
6	Pre-weaned
7	1–3 months
8	Pre-weaned
9	Pre-weaned
10	Pre-weaned

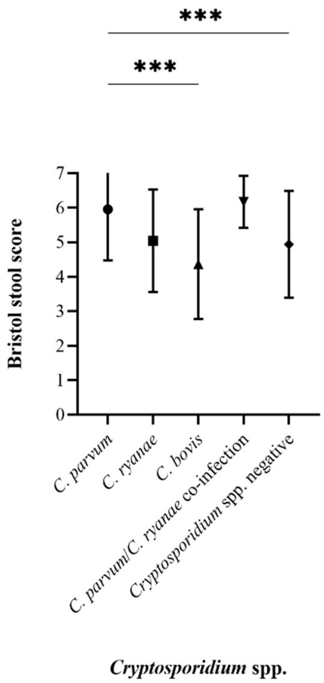

Figure S1. Bristol stool score ranges for the *Cryptosporidium* spp. identified in this study. Mean stool score for each species indicated by symbol - samples positive for *C. parvum* (●), C. *ryanae* (■), *C. bovis* (▲), *C. parvum*/*C.ryanae* co-infection (▼), and *Cryptosporidium*-negative samples (◆). Higher scores indicate softer, watery stools. Asterisks indicate statistically significant (Kruskal-Wallis analysis of variance with Dunn's pairwise comparisons test) differences in stool score between *Cryptosporidium* spp. Stool score for samples positive for *C. parvum* were significantly ($p < 0.001$) higher than samples positive for *C. bovis* and *Cryptosporidium*-negative samples. No other differences were statistically significant.

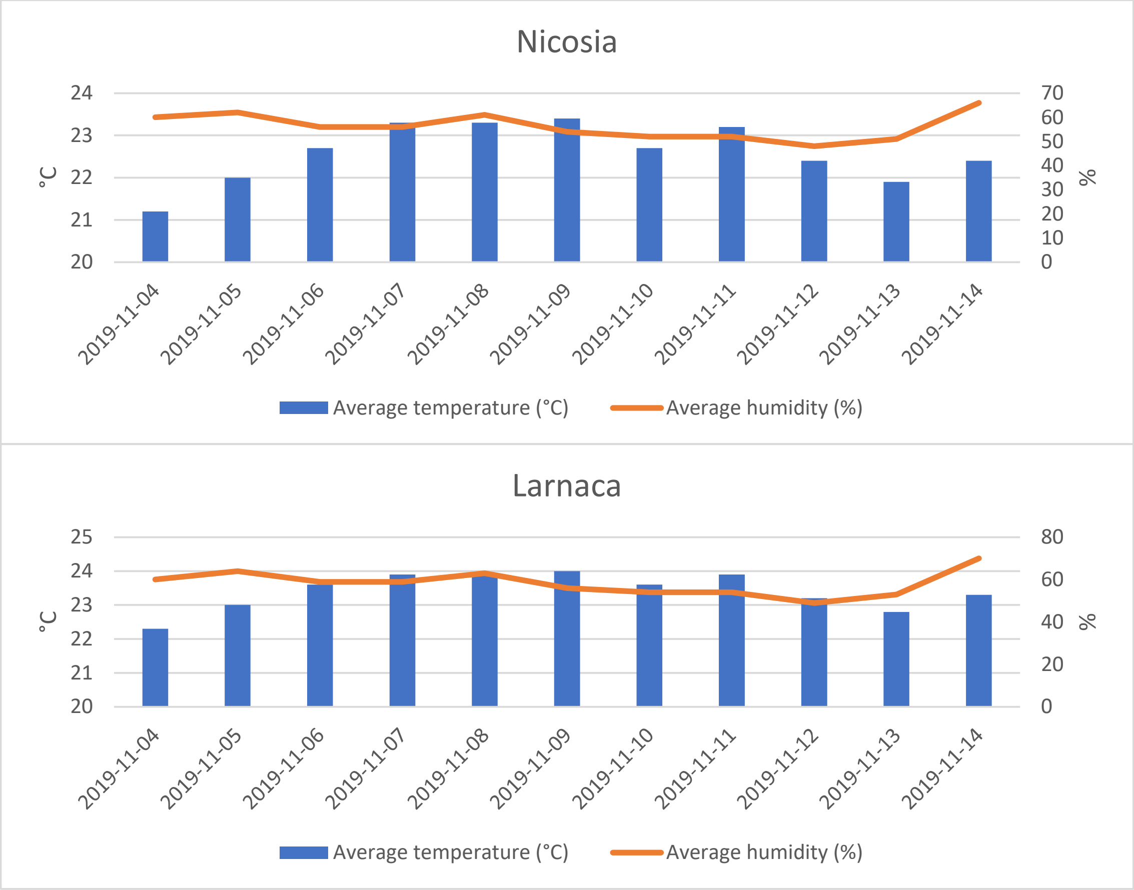

Figure S2. Meteorological report on Nicosia and Larnaca, indicating average temperature and rainfall in these regions during the sampling period. Stool samples were collected on the 7th, 8th and 14th of November 2019.
